# Comparing generative and extractive approaches to information extraction from abstracts describing randomized clinical trials

**DOI:** 10.1186/s13326-024-00305-2

**Published:** 2024-04-23

**Authors:** Christian Witte, David M. Schmidt, Philipp Cimiano

**Affiliations:** Semantic Computing Group, Center for Cognitive Interaction Technology, Bielefeld University, Inspiration 1, Bielefeld, 33619 NRW Germany

**Keywords:** Information Extraction, Deep Learning, Clinical Trials, Evidence-Based Medicine, PICO

## Abstract

**Background:**

Systematic reviews of Randomized Controlled Trials (RCTs) are an important part of the evidence-based medicine paradigm. However, the creation of such systematic reviews by clinical experts is costly as well as time-consuming, and results can get quickly outdated after publication. Most RCTs are structured based on the Patient, Intervention, Comparison, Outcomes (PICO) framework and there exist many approaches which aim to extract PICO elements automatically. The automatic extraction of PICO information from RCTs has the potential to significantly speed up the creation process of systematic reviews and this way also benefit the field of evidence-based medicine.

**Results:**

Previous work has addressed the extraction of PICO elements as the task of identifying relevant text spans or sentences, but without populating a structured representation of a trial. In contrast, in this work, we treat PICO elements as structured templates with slots to do justice to the complex nature of the information they represent. We present two different approaches to extract this structured information from the abstracts of RCTs. The first approach is an extractive approach based on our previous work that is extended to capture full document representations as well as by a clustering step to infer the number of instances of each template type. The second approach is a generative approach based on a seq2seq model that encodes the abstract describing the RCT and uses a decoder to infer a structured representation of a trial including its arms, treatments, endpoints and outcomes. Both approaches are evaluated with different base models on a manually annotated dataset consisting of RCT abstracts on an existing dataset comprising 211 annotated clinical trial abstracts for Type 2 Diabetes and Glaucoma. For both diseases, the extractive approach (with flan-t5-base) reached the best $F_1$ score, i.e. 0.547 ($\pm 0.006$) for type 2 diabetes and 0.636 ($\pm 0.006$) for glaucoma. Generally, the $F_1$ scores were higher for glaucoma than for type 2 diabetes and the standard deviation was higher for the generative approach.

**Conclusion:**

In our experiments, both approaches show promising performance extracting structured PICO information from RCTs, especially considering that most related work focuses on the far easier task of predicting less structured objects. In our experimental results, the extractive approach performs best in both cases, although the lead is greater for glaucoma than for type 2 diabetes. For future work, it remains to be investigated how the base model size affects the performance of both approaches in comparison. Although the extractive approach currently leaves more room for direct improvements, the generative approach might benefit from larger models.

## Introduction

The number of publications describing Randomized Controlled Trials has been increasing at an exponential pace for decades [1], thus making it more and more challenging to appropriately summarize the existing clinical evidence by way of systematic reviews. Yet, the ability to summarize the current clinical evidence is a core process to support evidence-based medical decision making [2]. Indeed, the creation of systematic reviews is costly and time consuming as it is done manually by clinical experts with the result that systematic reviews and guidelines quickly become outdated after publication or are even outdated at the time of publication [3–6]. Due to the effort associated with the creation of systematic reviews, there has been significant interest on the question how to automate their creation [7–9]. Recently, approaches to automatically summarize clinical evidence by way of argumentative structures have been proposed [10]. The bottleneck for such approaches is the missing availability of a database of semantically described clinical trials that comprise of structured representations of the key outcomes of each study. As argued by Sánchez-Graillet et al. [10], information extraction approaches have the potential to support the extraction of key information about the design and results of clinical trials from publications. These structured representations of the results of a trial in turn could support the process of systematic review creation or at least considerably reduce the effort to do so.

Most RCTs follow the PICO (**P**atient, **I**ntervention, **C**omparison, **O**utcomes) framework for structuring the presentation of clinical research findings. As a result, early IE approaches in the clinical domain classify full sentences of RCTs [11, 12] or smaller text spans [13] into the elements of the PICO framework. However, treating the PICO elements as flat objects represented as a collection of text spans does not reflect the complex information presented in RCTs for the following reasons: 1) the description of a single PICO element could be spread across several sentences and 2) the relationship between different PICO elements is not modelled (e.g. which outcomes belong to the intervention group and which ones belong to the comparison group).

Witte and Cimiano [14] have proposed an extractive information extraction approach that captures the design and key results of an RCT by way of 10 different templates that capture the PICO elements in a structured way, modelling dependencies and relations between them. These templates are based on the C-TrO Ontology that has been designed to support use cases related to the aggregation of evidence from multiple clinical trials [15]. Those templates are instantiated with information from a given abstract describing the trial. For instance, a template Medication with slots DrugName, DoseValue and DoseUnit could be used to describe medications of intervention arms mentioned in a RCT. However, Witte and Cimiano [14] assume that the number of template instances (e.g. number of outcomes) is provided a-priori, which hinders the application of their approach in real world settings. Further, the approach of Witte and Cimiano [14] chunks the text into smaller segments and then combines the templates instantiated for each segment. This makes it difficult to capture relations that are mentioned across chunks.

In this paper, we build on the approach of Witte and Cimiano [14] and extend it in two directions. First, we rely on Longformers [16] and Flan-T5 [17] in order to encode the complete abstract, inferring template instances and slots jointly for the complete text. Second, overcoming the key assumption that the number of template instances are known a priori, we extend the approach by a clustering step that induces the number of template instances in an unsupervised manner.

Beyond the extractive approach, we also present a generative approach that is inspired in recent seq2seq architectures such as REBEL [18] or GenIE [19]. These approaches rely on an encoder-decoder architecture by which the text is encoded and certain output structures are generated. We apply this idea to directly decode a complex nested template structure representing the design and key results of a study. As main novelties, we propose a decoding approach that relies on a grammar to guide decoding, ensuring that only valid structures are generated. Second, we present an approach to linearize the structure to be predicted such that it can be encoded as a sequence to be predicted by the generative approach. Our grammar-constrained decoding approach is inspired by Lu et al. [20], who also prune/mask the vocabulary to consist only of elements which comply with the desired output format. The decoding mechanism presented in this work generalizes the output format specification to arbitrary right-linear context-free grammars.

We evaluate and compare both approaches on the dataset provided by Sanchez-Graillet et al. [21] and used in previous work [14], which consists of predicting 10 templates. The dataset comprises a total of 211 documents for two diseases: type 2 diabetes (104) and glaucoma (107). Our results show that the improved extractive approach using Flan-T5 as a base model performs best for both diseases in the dataset, achieving a mean $F_1$ score of 0.547 ($\pm 0.006$) for type 2 diabetes and 0.636 ($\pm 0.006$) for glaucoma. However, both approaches have different strengths and weaknesses and are not yet suitable to fully automate the process of systematic review creation, but still have the potential to reduce the necessary effort a lot.

Additional data and evaluations (Appendix 2, 4 and 5) as well as the used grammar (Appendix 1) and a case study (Appendix 3) can be found in the appendix.

In summary, our contributions are the following:

- We present an extension of the approach proposed by Witte and Cimiano [14] in two directions: i) relying on Longformers [16] and Flan-T5 [17] to encode the complete abstract and infer templates and slots for the complete document jointly, and ii) using a clustering step to cluster the extracted template instances to infer the number of instances for each template type.
- We present a novel generative information extraction approach that relies on a grammar to guide decoding, and propose a novel serialization of the nested template structure such that the problem can be casted as a seq2seq inference problem.
- We evaluate both approaches on the dataset by Sanchez-Graillet et al. [21] and show that our improved extractive approach using Flan-T5 [17] as a base model performs best for both diseases.

## Related work

In recent years, a number of information extraction approaches have been developed, targeting tasks such as event extraction (e.g., Lu et al. [22], Hsu et al. [23], Yang et al. [20]), relation extraction (e.g., Giorgi et al. [24]) or role/slot/template filling (e.g. Du et al. [25, 26]). With respect to biomedical information extraction, there are also several approaches which aim to solve different tasks specifically for the domain of biomedical texts, e.g. scientific articles or clinical trials. Application domains range from event extraction (e.g., Wang et al. [27], Ramponi et al. [28], Zhu and Zheng [29], Huang et al. [30], Trieu et al. [31]) over relation extraction (e.g., Jiang and Kavuluru [32, 33]) and question answering (e.g., Wang et al. [27]) through to named entity recognition (e.g., Stylianou et al. [34]).

The set of methods and tools used to solve these problems is quite diverse, comprising joint end-to-end transformer models (e.g., Ramponi et al. [28], Trieu et al. [31], Jiang and Kavuluru [32], Stylianou et al. [34]) as well as support vector machines (e.g., Kim and Meystre [33]), conditional random fields (e.g., Stylianou et al. [35], Farnsworth et al. [34], Tseo et al. [36]), hybrid deep neural networks (e.g., Zhu and Zheng [29]) and Long Short-Term Memory networks (LSTMs, e.g., Jiang and Kavuluru [32], Kim and Meystre [33], Farnsworth et al. [35]).

Some related work also deals with detecting clinical trial outcomes, outcome spans (e.g., Abaho et al. [37–39], Ganguly et al. [40]) or slot fillers (e.g., Papanikolaou et al. [41]) in (randomized) clinical trial abstracts. However, they lack the specific structure and dependencies of PICO templates and slots, which are used in this paper. These approaches mostly use transformer architectures, sometimes in combination with, e.g., LSTMs to detect the outcomes/slot fillers.

The PICO framework is frequently used to describe the results of RCTs in a structured way. This structure comprises of a number of templates and corresponding slots (which are uniquely assigned to a single template type). However, a RCT can contain multiple instances of a template, imposing the problem of matching recognized slot fillers with their corresponding template instance.

Some efforts in this area focus on the problem that larger amounts of training data are missing or at least expensive to create due to the need for clinical experts as annotators. These approaches therefore utilize distant or weak supervision for training on noisy label data (e.g., Dhrangadhariya and Müller [42], Nye et al. [43], Wallace et al. [44], Liu et al. [45]). In contrast, the approach presented in this paper relies on the availability of sufficient classical supervised training data.

Other methods work with Conditional Random Fields (CRFs) in combination with (Bi-)LSTMs (e.g., Jin and Szolovits [46], Kang et al. [47]) or rule-based methods (e.g., Chabou and Iglewski [48]).

While most recent work relies on transformer architectures, there are also diverse other approaches which utilize different machine learning techniques like support vector machines (e.g., Yuan et al. [49]), convolutional neural networks (e.g., Stylianou et al. [50]), LSTMs (e.g., Jin and Szolovits [51]) or other deep learning-based approaches (e.g., Afzal et al. [52]).

Several recent approaches use transformer models like BERT (Bidirectional Encoder Representations from Transformers, Devlin et al. [53]) for PICO recognition, but focus on different architectual and task-related details.

However, some approaches refer to PICO elements as flat classes, i.e. parts of sentences are just labeled, e.g., P or I, whereas our approach considers PICO elements to be nested structures, i.e. templates with slots that have to be filled with some portion of text. Examples for this simplified view on PICO elements are listed in the following:

Schmidt et al. [54] treat the PICO recognition task as a sentence classification/question answering task and thus, in contrast to the approach presented in this paper, do not work on the level of whole documents/abstracts or PICO elements which span multiple sentences. Therefore, Schmidt et al. [54] do not benefit from contextualized representations utilizing the whole abstract as a context. Moreover, the problem of mapping found PICO elements to unique template instances is not dealt with.

Zhang et al. [55] propose a multi-step approach that first identifies P, I/C and O elements in the text using either Convolutional Neural Networks (CNNs) or Bi-LSTMs. After that, a Diseases Named Entity Recognition model is used to extract disease-related entities in the PICO-labeled sentences. Various different models, like, e.g., BERT-based or LSTM-based models, are compared in this category. Finally, a mapping model resolves some ambiguities, like intersections of recognition results for P and O. Again, different models (including both BERT and Bi-LSTMs) are evaluated for this task. Although this approach makes some efforts to create more structured results than flat sentence classification, it still ignores some aspects of the more complex structure of PICO elements.

Whitton and Hunter [56] propose a more structured view on PICO elements, e.g., by differentiating between two arms of a RCT. This is achieved in two steps by first applying a named-entity recognition model, recognizing three general types of entities (interventions, outcomes and measures). In a second step, they are then related to each other using a relation extraction model which also differentiates between the (up to) two arms of the considered RCTs. However, they focus on evidence tables, which are different from the nested template structure we work with in this paper. Moreover, the other approach does not work in a sequence-to-sequence manner with constrained decoding like the generative approach described in this paper.

Dhrangadhariya et al. [57] implement PICO recognition for more fine-grained entities, which - similarly to our approach - also consider more detailed information about participants, interventions and outcomes, like sample size, age, mortality, drugs or surgical interventions. Nevertheless, it is still less detailed than the template structure used in this paper, which consists of 10 templates comprising overall 85 slots (see Witte and Cimiano [14]). Moreover, by using BERT as an encoder and Bi-LSTM, self-attention as well as CRF and linear layers for classification, it does not work in a sequence-to-sequence manner like the generative approach we present in this work.

## Methods

**Fig. 1:**
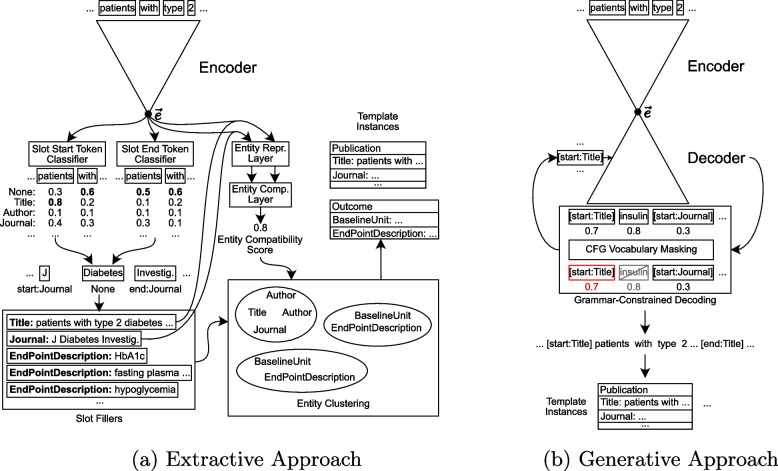
Illustration of both described approaches starting with the tokenized input and ending with the generated template instances

In this work, we address the problem of extracting a set of template instances from unstructured text. We tackle this problem from two different perspectives and present two approaches solving the same problem: 1) an extractive approach and 2) a generative approach. An illustration of both approaches can be found in Fig. 1.

**Fig. 2:**
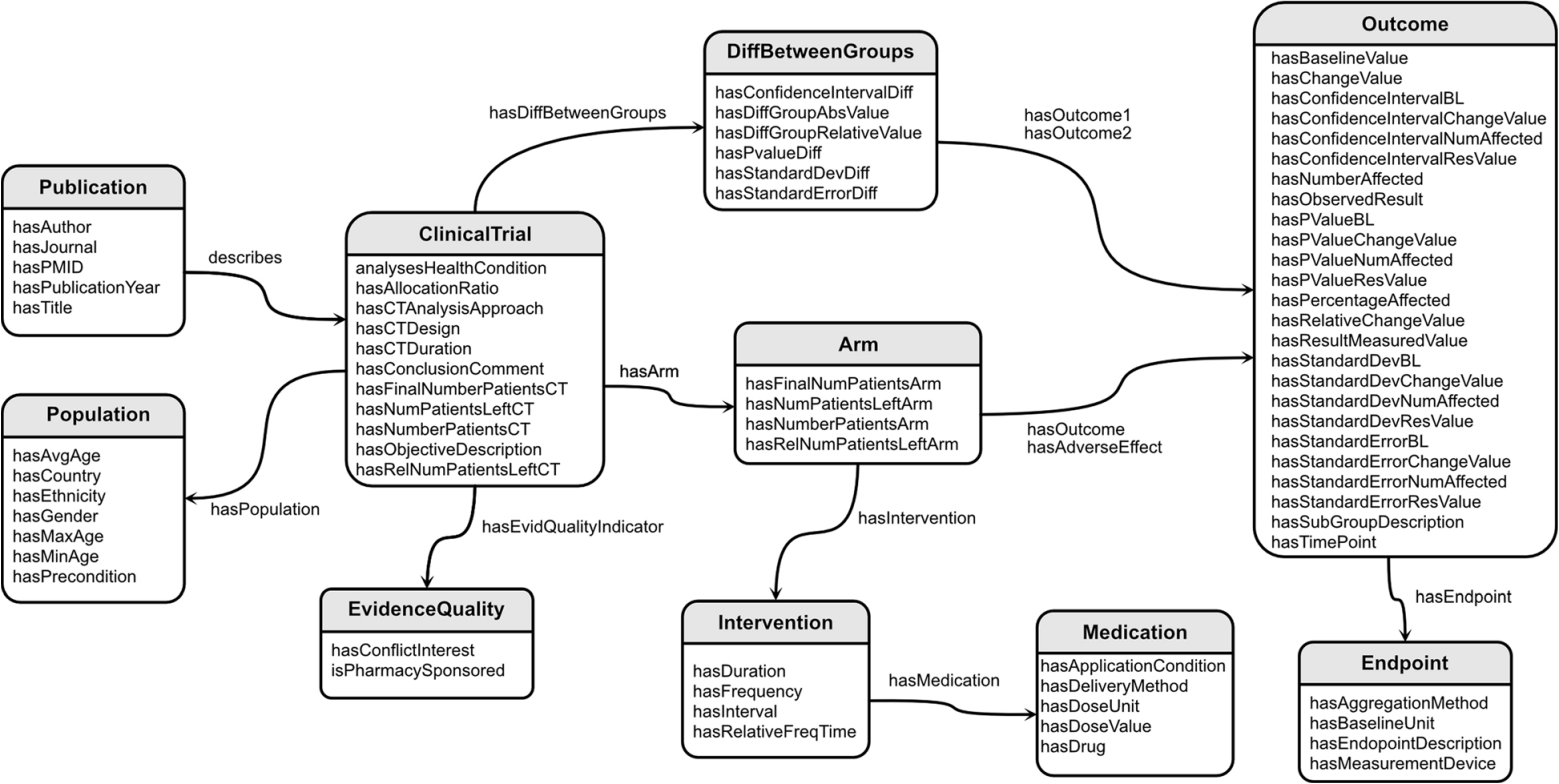
Schema of the PICO data model used in the experiments

The used data model captures the design and key results of an RCT by way of 10 different templates consisting of a total of 85 different slots that capture various aspects of the PICO elements in a structured way. These templates are based on the C-TrO Ontology that has been designed to support use cases related to the aggregation of evidence from multiple clinical trials [15]. The mean number of slot fillers per template is shown in Table 1. A template $t_i$ is defined by a type $i \in \mathcal L$ and a set of slots $\mathcal S_i = \bigcup _j s_{ij}$, where $s_{ij}$ denotes slot *j* of template $t_i$, $\bigcup _j$ this way denotes the set union over all slots *j* and $\mathcal L$ denotes the set of all template types. A template is instantiated by assigning slot-fillers to its slots, where a slot-filler can be either a text span from the input document or a template instance, depending on the slot. Figure 2 visualizes the used data model. In the following subsections, we describe the extractive and the generative approach in more detail.

**Table 1:** Mean and standard deviation of the number of slot fillers per template in the used dataset, separated by type of disease. Numbers rounded to two decimal places

Template	Type 2 diabetes	Glaucoma
Arm	7.01 ($\pm 2.79$)	4.8 ($\pm 2.07$)
ClinicalTrial	14.63 ($\pm 3.07$)	15.1 ($\pm 3.12$)
DiffBetweenGroups	3.61 ($\pm 0.81$)	3.32 ($\pm 0.72$)
Endpoint	1.68 ($\pm 0.85$)	1.81 ($\pm 0.95$)
EvidenceQuality	1.00 ($\pm 0.00$)	4.00 ($\pm 0.00$)
Intervention	1.91 ($\pm 0.79$)	2.24 ($\pm 0.74$)
Medication	1.98 ($\pm 1.11$)	2.13 ($\pm 1.17$)
Outcome	2.53 ($\pm 1.14$)	3.45 ($\pm 1.62$)
Population	3.13 ($\pm 1.86$)	2.32 ($\pm 1.09$)
Publication	12.45 ($\pm 3.44$)	10.14 ($\pm 3.79$)

### Extractive approach

Our extractive approach is based on the Intra-Template Compatibility (ITC) approach [14], which adopts a two-step architecture: In a first step, all textual slot-fillers are extracted from the input document, followed by a second step, which assigns the extracted slot-fillers to template instances. The extraction of slot-fillers and their clustering and assignment are described in the “Extraction of textual slot-fillers” and “Assignment of textual slot-fillers to template instances” Sections, respectively.

#### Encoding of the input document

The ITC approach uses BERT (Bidirectional Encoder Representations from Transformers) [53] to compute a contextualized representation of each token $w_i$ of the input document $d=(w_1,\ldots ,w_n)$. As the length of RCT abstracts typically exceeds the maximum number of tokens of most BERT implementations, the authors of ITC split the document into consecutive chunks and process each chunk separately. However, this approach treats each chunk as an isolated unit and hence the model is not able to learn token representations which incorporate the context of the full input document. Therefore, we adopt the Longformer [16] approach as well as the Flan-T5 model [17] to learn full-document contextualized representations $\textbf{h}_i \in \mathbb R^d$ (with $d = 768$ for both T5 and Longformer models) for each token $w_i$ of the input document, where *d* is the output dimension of the encoder of the respective model.

#### Extraction of textual slot-fillers

The ITC approach extracts slot-fillers from the input document by predicting start and end tokens of slot-fillers, followed by a step which joins the predicted start and end tokens. This is realized by training two linear layers which take the contextualized representation $\textbf{h}_i$ of the tokens $w_i$ as input and predicts whether or not this is a slot-filler start or end token, respectively:

1 $$\begin{aligned} \textbf{p}_{s,i} = \text {softmax}(\textbf{W}_s \textbf{h}_i + \textbf{b}_s) \qquad \textbf{W}_s \in \mathbb R^{|\mathcal S| \times d}, \quad \textbf{b}_s \in \mathbb R^{|\mathcal S|} \end{aligned}$$

2 $$\begin{aligned} \textbf{p}_{e,i} = \text {softmax}(\textbf{W}_e \textbf{h}_i + \textbf{b}_e) \qquad \textbf{W}_e \in \mathbb R^{|\mathcal S| \times d}, \quad \textbf{b}_e \in \mathbb R^{|\mathcal S|} \end{aligned}$$

where $\mathcal S = \bigcup _i \mathcal S_i \cup \{\mathbb O\}$ is the set of all slots including the special no-slot label $\mathbb O$ which indicates that a token is not classified as a start/end token of a slot-filler. The vectors $\textbf{p}_{s,i}$, $\textbf{p}_{e,i}$ denote the predicted probability distribution over the slots that a token $w_i$ is the start/end of the respective slots. The final prediction is determined by the $\arg \max$ operation.

The predicted start/end tokens are joined sentence-wise by minimizing the distance between start and end tokens in terms of tokens in between. More precisely, for a given sentence, we first collect all predicted start and end tokens. For each predicted start token $w_s$, at position *i* we seek an end token $w_e$ at position $j \ge i$ with matching label and minimal distance to $w_s$ and assign it to $w_s$ as its end token. Finally, we discard predicted start/end tokens which have no matching end/start token. This slightly differs from the IOB format [58], as only start and end token of a sequence are tagged and all tokens in between are classified just like tokens which are not part of any sequence. A comparison of both tagging schemes can be found in Table 2.

**Table 2:** Comparison of used tagging schema with the IOB format, where O represents tokens outside of a sequence and I-Frequency represents tokens which are part of a slot filler sequence of type frequency. In contrast, None represents tokens which are neither start nor end token of a slot filler, start:Frequency marks the start and end:Frequency the end of a frequency slot filler sequence

Tokens	NPH	insulin	once	or	twice	daily	in
**IOB**	O	O	I-Frequency	I-Frequency	I-Frequency	I-Frequency	O
**Used**	None	None	start:Frequency	None	None	end:Frequency	None

For each extracted slot-filler *i* with start/end tokens $w_s$ resp. $w_e$ with corresponding token representations $\textbf{h}_s$, resp. $\textbf{h}_e$, ITC computes a representation $\textbf{e}_i$ by summing the representations of the start and end tokens followed by a dense layer with ReLU [59] activation function:

3 $$\begin{aligned} \textbf{e}_i = \text {relu}(\textbf{W}_r(\textbf{h}_s + \textbf{h}_e) + \textbf{b}_r) \qquad \textbf{W}_r \in \mathbb R^{d \times d}, \quad \textbf{b}_r \in \mathbb R^d \end{aligned}$$

The learned representations $\textbf{e}_i$ of the extracted slot-fillers (SFs) are then used as input to subsequent modules. In the remainder of this paper, we denote the set of all extracted slot fillers as $\mathcal E$, where each slot filler in $\mathcal E$ is represented by its vector representation computed by Eq. (3).

#### Assignment of textual slot-fillers to template instances

Typically, for some slot types like the textual slot fillers of the Outcome template, there are several slot fillers of the same type extracted from an original document. Therefore, we need a way to group these slot fillers such that actual template instances, e.g. multiple Outcome instances, can be created from these slot fillers. Deciding which slot fillers belong together is however not a trivial task.

The assignment of extracted SFs to template instances is therefore done in ITC by a clustering approach per template based on a pairwise similarity or compatibility function $q: \mathbb R^d \times \mathbb R^d \rightarrow [0, 1]$. *q* scores the similarity between two SFs in the sense that they belong to the same template instance, where $g(\textbf{e}_i, \textbf{e}_j)=1$ indicates maximum similarity such that $\textbf{e}_i$ and $\textbf{e}_j$ should be assigned to the same template instance. Note that $\textbf{e}_i$ and $\textbf{e}_j$ are entity representations calculated based on the contextualized embeddings generated by the used models. Thus, we can use results from the established field of (density-based) clustering to figure out the SF grouping. The similarity function *q* is implemented in a slightly more complex way compared to the original paper, using two linear layers with a ReLU activation function in between and followed by a sigmoid activation function:

4 $$\begin{aligned} q'(\textbf{e}_i, \textbf{e}_j){} & {} = \text {relu}(\textbf{W}_h(\textbf{e}_i + \textbf{e}_j) + \textbf{b}_h)\quad \quad \textbf{W}_h \in \mathbb R^{d \times d}, \quad \quad \textbf{b}_h \in \mathbb R^d \end{aligned}$$

5 $$\begin{aligned} q(\textbf{e}_i, \textbf{e}_j){} & {} = \sigma (\textbf{w}_s^T(q'(\textbf{e}_i, \textbf{e}_j)) + \textbf{b}_s)\quad \quad \ \ \textbf{w}_s \in \mathbb R^d, \quad \qquad \ \ \textbf{b}_s \in \mathbb R \end{aligned}$$

Note that due to the symmetry of $+$, also *q* is a symmetric function, i.e. $q(\textbf{e}_i, \textbf{e}_j) = q(\textbf{e}_j, \textbf{e}_i)$ for all pairs of $\textbf{e}_i, \textbf{e}_j$. Then the mean pairwise similarity between SFs of a cluster $C_i \subseteq \mathcal E$ is given by

6 $$\begin{aligned} g(C_i) = \frac{1}{|C_i \times C_i|} \sum \limits _{(\textbf{e}_i, \textbf{e}_j) \in C_i \times C_i} q(\textbf{e}_i, \textbf{e}_j) \end{aligned}$$

The score of a clustering $\mathbb C_i = \{ C_1, \ldots , C_{m_i} \}$ of SFs $\mathcal E_i \subseteq \mathcal E$ for template $t_i$ is the mean score of its cluster scores:

7 $$\begin{aligned} h(\mathbb C_i) = \frac{1}{|\mathbb C_i|} \sum \limits _{C_k \in \mathbb C_i} g(C_k) \end{aligned}$$

The ITC approach seeks a clustering $\mathbb C_i^*$ of $m_i$ clusters which maximizes the score given by Eq. (7):

8 $$\begin{aligned} \mathbb C_i^* (m_i) = \arg \max _{\mathbb C_i \in \mathcal U_{i,m_i}} h(\mathbb C_i) \end{aligned}$$

where $\mathcal U_{i,m_i}$ denotes the set of all clusterings of the set $\mathcal E_i$ with $m_i$ clusters. Note that the optimization objective defined by Eq. (8) is parameterized by the number of clusters $m_i$. In order to alleviate the assumption that the number of instances of templates needs to be known a priori, we propose a clustering step to induce the number of template instances per template type using Hierarchical Agglomerative Clustering (HAC) with a threshold based on the average of values computed for the training data, namely:

- the average similarity values of pairs belonging to the same template instance
- the average similarity values of pairs belonging to different instances

After the clustering $\mathbb C_i^*(m_i)$ has been estimated, the template instances $t_{ij}$ are derived from those clusters $C_j^* \in \mathbb C_i^*(m_i)$. The slot to which a SF $\textbf{e}_k \in C_j^*$ is assigned is given by the label assigned by the SF extraction module by Eqs. (1) and (2). In summary, the assignment of SFs to template instances is done as follows:

1. For each template $t_i$, the set $\mathcal E_i \subseteq \mathcal E$ of SFs which can be assigned to instances of template type $t_i$ is estimated.
2. Equation (8) or Agglomerative Hierarchical Clustering is used to find some clustering of the SFs in $\mathcal E_i$.
3. The template instances are derived from the clusters in the clustering.

As an example, we consider the following four extracted slot fillers:

1. PercentageAffected: 16
2. PercentageAffected: 8
3. TimePoint: week 24
4. TimePoint: week 12

Additionally, we assume our trained similarity function gives us the similarities presented in Table 3.

**Table 3:** Example similarities/compatibilities between four slot fillers, slot types in first row have been omitted

	16	8	week 24	week 12
PercentageAffected: 16	-	0.1	0.7	0.4
PercentageAffected: 8	0.1	-	0.3	0.8
TimePoint: week 24	0.7	0.3	-	0.2
TimePoint: week 12	0.4	0.8	0.2	-

Given these similarities and a clustering threshold of, e.g., 0.5, this results in two clusters which can be then directly used to create the corresponding Outcome template instances. These two clusters are:

1. PercentageAffected: 16 and TimePoint: week 24
2. PercentageAffected: 8 and TimePoint: week 12

The clustering thus provides a robust and flexible way to both determine the number of template instances to generate as well as the groups of slot fillers those instances comprise.

### Generative approach

In this section we propose a simple generative approach for extracting template instances from unstructured text based on the Transformer [60] encoder-decoder model. As encoder-decoder models require the output to be a linear token sequence, the set of TIs needs to be converted into a sequence of tokens. In Section “Linearization of sets of template instances”, we present a simple recursive method for linearizing sets of TIs along a context free grammar (CFG) for describing the linearized structures. In Section “Decoding” we adopt the presented CFG for generating valid token sequences representing sets of TIs.

#### Transformer-based encoder-decoder models

Transformer-based [60] encoder-decoder models are seq2seq models which haven been used on a variety of natural language processing tasks like machine translation [61] and text summarization [62]. The encoder part of the Transformer learns a contextualized representation of the input tokens $w_1, \ldots , w_n$ via multi-headed self-attention [60], converting the input sequence into a sequence of vectors $\textbf{h}_1, \ldots , \textbf{h}_n \in \mathbb R^d$, where *d* is the dimension of the Transformer model. Then the decoder part takes the vector sequence from the encoder as input and produces an output vector sequence $\textbf{d}= (\textbf{d}_1, \ldots , \textbf{d}_n \in \mathbb R^d)$ via multi-headed cross-attention. The computational complexity of self-attention grows quadratically with the number of tokens. Beltagy et al. [16] proposed the Longformer encoder-decoder, which combines local and global multi-headed self-attention in the encoder, reducing computational complexity from $\mathcal O(n^2)$ to $\mathcal O(n)$.

The output vector sequence $\textbf{d}$ is used to compute a probability distribution over the vocabulary of the underlying model via the following equation:

9 $$\begin{aligned} p(y_i | x, y_1, \ldots , y_{t-1} ) = \text {softmax} (\textbf{v}_i^T \textbf{d}_{t-1} + b_i) \end{aligned}$$

where $\textbf{v}_i \in \mathbb R^d$ is the embedding of token $y_i$, $b_i$ is a bias for token $y_i$, $\textbf{d}_{t-1}$ is the output vector of the decoder at position $(t-1)$ and *d* is the model dimension. The probability of token $y_t$ at position *t* is conditioned on the input token sequence *x* and the past decoded tokens $y_1, \ldots , y_{t-1}$. This dependence is encoded through the vector $\textbf{d}_{t-1}$ via multi-headed self- and cross-attention.

Token prediction in the decoder is done by maximum a posteriori probability (MAP) inference. Hence the predicted token at position *i* is given by the token with maximal posterior probability:

10 $$\begin{aligned} y_t = \arg \max _i p(y_i | x, y_1, \ldots , y_{t-1} ) \end{aligned}$$

The generative model is trained via teacher forcing by minimizing the cross entropy loss between the predicted token distribution described by Eq. (9) and the ground truth label.

#### Linearization of sets of template instances

As encoder-decoder models expect the output space to be token sequences, we present a simple recursive linearization procedure of template instances (TIs). First, note that TIs are described by the content of their slots (i.e., their slot-fillers), and that slot-fillers can be either text spans from the input document or other TIs. Hence the recursion base is given by the linearization of textual slot-fillers. Let $f = w_{k_1}, \ldots , w_{k_m}$ be a token sequence which represents a textual slot-filler *f* for a slot of name SLOT. Then the linearization of this slot-filler is the token sequence itself enclosed by the special tokens [start:SLOT] and [end:SLOT], i.e. [start:SLOT] $\odot ~w_{k_1} \odot \ldots \odot w_{k_m} \odot$ [end:SLOT], where $\odot$ denotes the concatenation of tokens. If the slot-filler is a TI, then it is recursively linearized and the resulting token sequence is enclosed by the special tokens [start:SLOT] and [end:SLOT]. The linearization of TIs is described below.

In general, more than one slot-filler can be assigned to a slot of a TI. Therefore, we denote the complete content of a slot as a set $\mathcal F$ of slot-fillers. As sets, in contrast to sequences, are unordered constructs by definition, the linearization of sets of slot-fillers is inherently ambiguous. To get an unambiguous order, we introduce a slot ordering operator $\omega$ which converts sets of slot-fillers into sequences of slot-fillers according to predefined criteria (e.g. position within input document in case of textual slot-fillers). Then sets $\mathcal F$ of slot-fillers are linearized as follows: First, we sort the elements of $\mathcal F$ according to the sorting operator $\omega$ and obtain a sequence *F* of slot-fillers. Then we linearize each slot-filler in *F* as described above and concatenate the resulting token sequences, respecting the ordering of slot-fillers in *F*.

Next, we describe the linearization of TIs. As TIs are represented by the content of their slots, the linearization of a TI has to include the linearization of its slots. However, a template does not impose any ordering of its slots, and hence the linearization order of the slots of a TI is undefined. Therefore, we introduce another ordering operator $\Omega$ which orders the slots of a template. Then the linearization of a TI is the concatenation of the linearizations of its slots according to the ordering of its slots given by the ordering operator $\Omega$.

Any set of TIs induces a graph with TIs as nodes and links between TIs as edges. Recall that there is a link from TI $t_{ij}$ to TI $t_{kl}$ iff $t_{kl}$ is a slot-filler of $t_{ij}$. In order to guarantee that the linearization algorithm described above is well defined, we require the induced graph to be 1) acyclic and 2) connected. The first requirement ensures that the linearization algorithm terminates, while the second ensures the absence of isolated TI, which can not be linearized.

However, choosing $\omega$ and $\Omega$ is only necessary for training but not for inference purposes, as the decoding allows to fill template slots in any order. Therefore, we choose arbitrary but fixed $\omega$ and $\Omega$ for the experiments described in the “Experimental results” Section.

**Fig. 3:**
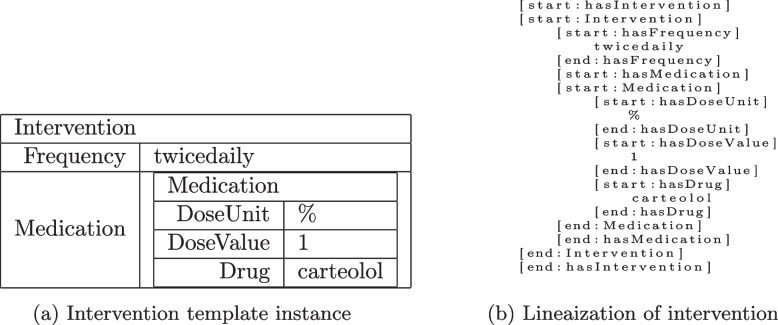
Illustration of linearization of an intervention template instance

A full example for a whole linearized publication template instance can be found in Listing 2 in Appendix 6. A shorter example for an intervention template instance with both textual and template slot fillers can be found in Fig. 3.

#### A context-free grammar for describing linearization of sets of template instances

In the following, we describe the linearization of sets of TIs (described in Section “Linearization of sets of template instances”) by a context-free grammar (CFG) which is used in the decoding process (“Decoding” Section) to constrain the generation of tokens. A CFG is defined by a 4-tuple $\mathcal G = (N, T, R, S)$, where *N* is a set of non-terminal symbols, *T* is a set of terminal symbols, *R* is a set of production rules and $S \in T$ is the start symbol of the grammar. The set of terminal symbols is defined by the vocabulary of the underlying encoder-decoder model together with some special tokens for defining the production rules *R*. The recursion base of the linearizations of sets of TIs is given by the linerization of textual slots which we describe by the following equation:

11 $$\begin{aligned} \texttt {TEMPLATE } := \texttt { [start:SLOT] TEXT [end:SLOT] TEMPLATE} \end{aligned}$$

where TEMPLATE and SLOT are placeholders for names of template and slots, respectively, TEXT is a placeholder for any token sequence from the input document and [start:SLOT], [end:SLOT] are special tokens enclosing the textual slot-filler. Eq. (11) schematically defines production rules for textual slot-fillers, and TEMPLATE is the non-terminal symbol which is used to identify the respective production rules. Note that the non-terminal symbol TEMPLATE on the right-hand side of Eq. (11) allows recursion and hence the application of more the one production rule associated with the non-terminal symbol TEMPLATE. The recursion base of production rules is given by

12 $$\begin{aligned} \texttt {TEMPLATE } := \texttt { [end:TEMPLATE]} \end{aligned}$$

where TEMPLATE is again a placeholder for the template name and [end:TEMPLATE] is a special token indicating the end of the linearization of the template TEMPLATE. Production rules for TIs are described by

13 $$\begin{aligned} \texttt {TEMPLATE\_HEAD } := \texttt { [start:TEMPLATE] TEMPLATE} \end{aligned}$$

Analogously to the production rules defined by Eq. (11) for textual slot-fillers, the production rules for slots containing TIs as slot-fillers is defined by

14 $$\begin{aligned} \texttt {TEMPLATE } := \texttt { [start:SLOT] TEMPLATE\_HEAD [end:SLOT] TEMPLATE} \end{aligned}$$

where TEMPLATE_HEAD is a placeholder for any non-terminal symbol whose associated production rules are derived from Eq. (13). Listing 1 shows the production rules for the data model used in our experiments.

### Decoding

In Section “A context-free grammar for describing linearization of sets of template instances”, we presented a CFG which describes valid token sequences representing a set of TIs. In this section, we describe a simple method to constrain token prediction such that only such token sequences are generated which are valid according the CFG. For example, consider a slot Drug which can have textual slot-fillers for describing drug names for a medication. After the special token [start:Drug] has been predicted, we know that the set of next possible tokens would consist of all tokens from the input document plus the special token [end:Drug]. This information is encoded by the CFG, and the decoding method described in this section uses this information to constrain token prediction.

In this paper, we slightly generalize the constrained decoding approach of Lu et al. [20] to arbitrary right-linear CFGs by applying a strategy similar to recursive descent parsing.

Beginning with a start symbol, in our case PUBLICATION_HEAD, the set of possible next tokens is calculated in each decoding step. This set is then used to generate a mask for the model vocabulary to discard all tokens which would not comply with the production rules of the CFG. From the remaining tokens, we select the token with the maximum value in a greedy fashion. The implementation of a beam search to optimize the decoding output even more remains for future work.

To keep track of the decisions and possible next tokens, a stack data structure is used to guide the decoding. Whenever a start token of a slot like [start:NumberAffected] is chosen as the decoded token, this decision is saved by adding this to the decoding stack. This is then used to constrain the tokens in the next step to be only those which can follow a [start:NumberAffected] token. Similarly, when an end token like [end:NumberAffected] is chosen, the top stack element is removed from the stack.

This way, the decoding is guided to comply with the requirements imposed by the CFG and this way ensuring the output can then be parsed into actual TIs.

## Experimental results

In this section, we discuss the setting of our experiments as well as the results of those experiments.

### Experimental setting

In our experiments, we use the same dataset as Witte and Cimiano [14] for type 2 diabetes and glaucoma. The dataset comprises a total of 211 documents for two diseases: type 2 diabetes (104) and glaucoma (107). The 104 type 2 diabetes documents are split up into a training, validation and test sets of size 68, 16 and 20, respectively. Analogously, the 107 glaucoma documents are split up into a training, validation and test sets of size 69, 17 and 21, respectively. We use the same fixed train-validation-test split and run separate experiments for those two diseases. Both the extractive and the generative approach were then evaluated using multiple base models, namely allenai/longformer-base-4096 [16] for the extractive approach and allenai/led-base-16384 [16] as well as google/flan-t5-base [17] for both approaches. As the extractive approach requires just an encoder whereas the generative approach needs a decoder due to its seq2seq nature, we compare two encoder-decoder models from which only the encoder is used in the extractive approach. Additionally, we also evaluate an encoder-only model for the extractive approach to ensure the partial usage of the encoder-decoder models does not harm the performance.

For these models and diseases, we then run hyperparameter optimizations using Optuna [63] with 30 trials each and measuring performance using validation $F_1$ scores. In each trial, an initial learning rate (between $1e^{-3}$ and $1e^{-5}$, using logarithmic domain) and a $\lambda$ for the lambda learning rate scheduler (between 0.9 and 1.0, using logarithmic domain, learning rate calculated with $lr ( epoch ) = \lambda ^ {epoch}$) are sampled from Optuna. The used batch size is 1 and the number of epochs is 50 in all experiments. Each experiment is then run on a single NVIDIA A40 GPU. The best hyperparameters for each disease-approach-model-combination are then used to train 10 additional models. Unless stated differently, mean and standard deviation in tables refer to the different results of these 10 training runs. The means and standard deviations of the test $F_1$ scores of these 10 trained models are listed in Table 4 for each combination.

### Slot-filler extraction results

In all categories, the extractive approach paired with the flan-t5-base model performs best. In summary, for glaucoma, the extractive approach performs best with model flan-t5-base and a mean test $F_1$ score of 0.636 ($\pm 0.006$ standard deviation across the 10 training runs with the best found hyperparameters of the category). This way, it outperforms the other tested models of the extractive approach as well as all models of the generative approach by 0.02 or more. For type 2 diabetes, the extractive approach performs best as well with model flan-t5-base and a mean $F_1$ score of 0.547 ($\pm 0.006$ standard deviation). This indicates that the extractive approach is superior to the generative approach, although the lead is much smaller for type 2 diabetes than for glaucoma.

Table 5 shows the mean $F_1$ scores per template on the type 2 diabetes and glaucoma test set. The table shows the values of the best models of each category (w.r.t. validation $F_1$ score), i.e. the flan-t5-base models in all four cases. The mean $F_1$ values are calculated for each of the 10 models trained using the best hyperparameters of their respective category. The values in the table correspond to the mean and standard deviation of those mean $F_1$ scores per template. The generative approach performs better than the extractive one on the Medication templates (0.48 vs. 0.34 and 0.62 vs. 0.53 $F_1$ score for type 2 diabetes and glaucoma, respectively). On the Population and Outcome template, the results are mixed with one approach performing better for one disease dataset but not for the other. On all six remaining templates, the extractive approach performs better, although with different margins.

Mean $F_1$ scores per slot are shown in Table 7 in the Appendix 2, again with mean and standard deviation (of the mean $F_1$ scores) calculated for the 10 models trained using the best hyperparameters of their respective category. The $F_1$ scores of the different slots range from over 0.9, e.g. PMID or PublicationYear, to below 0.1, e.g. FinalNumPatientsArm or ObservedResult. There are also some noticeable differences between the diseases, with Journal achieving scores of 0.96 and 0.92 for type 2 diabetes in contrast to 0.67 and 0.74 for glaucoma. There are also slots where one approach performs better than the other across both datasets, e.g. DoseUnit (0.77/0.8 generative vs. 0.24/0.6 extractive) and NumberPatientsCT (0.65/0.65 generative vs. 0.93/0.86 extractive).

#### Joint training on both datasets

Additionally to the main experiment described above, we ran another small experiment, training the best-performing generative and extractive model (flan-t5-base in both cases) with the best-performing respective parameters in 10 trials on the union of the type 2 diabetes and glaucoma training, validation and test datasets, respectively. The resulting models are then again evaluated on the separated datasets for comparability reasons. The resulting mean $F_1$ scores ($\pm \sigma$) for the generative approach are 0.556 (± 0.026) for type 2 diabetes and 0.626 (± 0.015) for glaucoma. For the extractive approach, the mean $F_1$ scores ($\pm \sigma$) are 0.560 (± 0.007) for type 2 diabetes and 0.644 (± 0.008) for glaucoma. Therefore, the performance increases for both datasets and both approaches compared to the original results trained on the separated datasets. Moreover, the generative approach achieves comparable performance to the scores of the extractive approach trained on the separated datasets. At the same time, the extractive approach gets even better when also trained on both datasets at the same time.

Considering the relatively small datasets, this might indicate that performance for both diseases benefits from similar data in the other dataset, respectively. Therefore, we are optimistic that the training of a single general model (in contrast to specialized models for each disease as described in the main experiment) is possible with comparable or even better performance on diseases the model has been trained on (i.e., in-distribution data) and acceptable performance on different but similar diseases (i.e., out-of-distribution data). However, another dataset would be necessary to test this hypothesis such that this remains to be investigated in future work.

### Inferred template cardinality results

In this section, we evaluate the ability of our models to infer the correct number of instances for each template type. For this, we compare the number of inferred templates to the number of instances in the gold standard by computing the mean abolsute deviation. Table 6 shows the mean absolute deviation between the ground truth and predicted template cardinality of the best extractive and generative model on the type 2 diabetes and glaucoma test sets. The mean absolute deviation values are calculated separately for each of the 10 models trained using the best hyperparameters of their respective category. The values in the table are then mean and standard deviation of those mean absolute deviations across the respective 10 trained models. Additionally, in Appendix 5, the corresponding mean ground truth (GT) and predicted template cardinalities are listed in order to allow a judgement whether or not a certain deviation is high. Note that the templates Publication, ClinicalTrial and Population are not mentioned in these tables as their cardinality is always one.

On the type 2 diabetes dataset, the extractive approach yields better results than the generative approach in terms of template cardinality prediction for the DiffBetweenGroups, Endpoint and Medication templates, whereas the generative approach yields better results for the Arm, Intervention and Outcome templates. On the glaucoma dataset, the generative approach performs better than the extractive one in terms of cardinality inference on all templates except DiffBetweenGroups (0.39 vs. 0.17) and Endpoint (2.91 vs. 0.35).

**Table 4:** Mean and standard deviation $\sigma$ of test $F_1$ scores across 10 models trained using best-performing ($F_1$ on validation dataset) configuration found in 30 trials of hyperparameter optimization. Numbers rounded to three decimal places, best configuration of each disease marked bold

Type 2 diabetes		Glaucoma	
Model	Mean $F_1$ ($\pm \sigma$)	Model	Mean $F_1$ ($\pm \sigma$)
Extractive			
**flan-t5-base**	**0.547 (± 0.006)**	**flan-t5-base**	**0.636 (± 0.006)**
led-base-16384	0.525 (± 0.009)	led-base-16384	0.572 (± 0.010)
longformer-base-4096	0.540 (± 0.008)	longformer-base-4096	0.613 (± 0.007)
Generative			
flan-t5-base	0.539 (± 0.029)	flan-t5-base	0.584 (± 0.025)
led-base-16384	0.400 (± 0.079)	led-base-16384	0.353 (± 0.106)

**Table 5:** Mean slot $F_1$ values per template. Each cell shows mean and standard deviation of 10 training runs with the best found hyperparameters for best (w.r.t. validation $F_1$ score) configurations of each category. Numbers rounded to two decimal places, best values marked bold

	Type 2 diabetes $F_1$ ($\pm \sigma$)		Glaucoma $F_1$ ($\pm \sigma$)	
Template name	Generative	Extractive	Generative	Extractive
Arm	0.7 (± 0.21)	**0.87 (± 0.02)**	0.34 (± 0.06)	**0.36 (± 0.04)**
ClinicalTrial	0.62 (± 0.02)	**0.82 (± 0.02)**	0.63 (± 0.03)	**0.78 (± 0.02)**
DiffBetweenGroups	0.41 (± 0.06)	**0.45 (± 0.03)**	0.28 (± 0.08)	**0.37 (± 0.04)**
Endpoint	0.39 (± 0.03)	**0.43 (± 0.01)**	0.33 (± 0.04)	**0.42 (± 0.09)**
Intervention	0.61 (± 0.06)	**0.62 (± 0.02)**	0.26 (± 0.02)	**0.42 (± 0.12)**
Medication	**0.48 (± 0.02)**	0.34 (± 0.02)	**0.62 (± 0.08)**	0.53 (± 0.02)
Outcome	**0.2 (± 0.03)**	0.11 (± 0.01)	0.35 (± 0.04)	**0.38 (± 0.01)**
Population	0.22 (± 0.03)	**0.52 (± 0.07)**	**0.56 (± 0.04)**	0.52 (± 0.03)
Publication	0.95 (± 0.03)	**0.96 (± 0.01)**	0.86 (± 0.02)	**0.9 (± 0.02)**

**Table 6:** Mean absolute deviation between ground truth and predicted template cardinality. Each cell shows mean and standard deviation of 10 training runs with the best found hyperparameters for best (w.r.t. validation $F_1$ score) configurations of each category. Numbers rounded to two decimal places, best values marked bold

	Type 2 diabetes		Glaucoma	
Template name	Generative	Extractive	Generative	Extractive
Arm	**0.01 (± 0.02)**	1.09 (± 0.03)	** 0.02 (± 0.04)**	1.33 (± 0.05)
DiffBetweenGroups	1.01 (± 0.96)	**0.69 (± 0.08)**	0.39 (± 0.36)	**0.17 (± 0.13)**
Endpoint	5.32 (± 1.11)	**3.83 (± 0.03)**	2.91 (± 1.09)	**0.35 (± 0.22)**
Intervention	**0.1 (± 0.04)**	1.22 (± 0.03)	**0.19 (± 0.05)**	0.8 (± 0.1)
Medication	0.21 (± 0.07)	**0.18 (± 0.1)**	**0.11 (± 0.08)**	0.47 (± 0.2)
Outcome	**1.08 (± 0.82)**	8.36 (± 0.07)	**0.94 (± 0.57)**	2.98 (± 0.04)

## Discussion

The overall slot-filler extraction results of both models in terms of micro $F_1$ measure indicate that the extractive approach is slightly superior to the generative approach, although the margin is especially small for the type 2 diabetes dataset (cf. Table 4). Moreover, the mean $F_1$ scores per template (Table 5) suggest that the extractive approach performs better than the generative one on most templates on both datasets.

However, the full picture is a little more complex and both approaches have areas in which they perform better or worse than the other one and vice versa, and that for a variety of reasons.

First, it is noticeable that the $F_1$ scores for glaucoma are, on average, higher than those for type 2 diabetes. Nevertheless, the difference between the results for both datasets is not the same for both approaches, although the trend is the same. For the generative approach, the performance of the best-performing flan-t5-base model decreases by just 0.045 (around $7.7\%$ relatively) and the led-base-16384 version even increases its mean performance.

In contrast, the best-performing extractive version, again flan-t5-base, loses 0.089 (around $14\%$ relatively) in terms of $F_1$ performance - relatively almost twice as much as the generative approach. This may indicate that the extractive approach is better able to exploit certain characteristics which are specific to the glaucoma dataset and which are not present in the type 2 diabetes dataset, whereas the generative approach is more robust against those differences - both in a positive and in a negative way - and that way maybe generalizing a little more due to the more complex nature of the seq2seq task. However, it is not clear which properties of the data cause this deviation.

Considering robustness and the different complexity of the tasks of the extractive and generative task, this is to some degree also mirrored by the standard deviations of the two approaches. While the standard deviation for the extractive approach is not greater than 0.01, the standard deviation of the generative models is not smaller than 0.025 and gets up to 0.106 for led-base-16384. Therefore, it is more than doubled at least compared to the extractive approach.

Moreover, the standard deviation appears to be correlated to the chosen model, with flan-t5-base giving the lowest deviation, followed by (for the extractive part) longformer-base-4096 and finally led-base-16384 consistently across both datasets.

The different strengths and weaknesses of both approaches become even more apparent examining the different performances separated by templates (Table 5) and, ultimately, single slots (Table 7 in the Appendix 2).

For whole templates, Table 5 shows an in parts mixed picture of which approach performs best. In many cases in which the extractive approach performs best, both approaches perform similarly well (e.g., Publication). However, there are also different cases like Clinical Trial where the margin is larger, but also Medication where the generative approach outperforms the extractive approach by around 0.1 although the standard deviation is also quite high for the generative glaucoma case. In other cases there are large differences between the two datasets, which is also true for the evaluation per slot.

As an example for unexpected single slot differences, consider the Journal slot. One would expect the recognition of the Journal slot to be a comparably simple task across both datasets. However, the performance greatly differs between the datasets, although both approaches achieve good scores on this slot. For the type 2 diabetes dataset, the performance is nearly perfect with scores above 0.9. In contrast, the scores for the glaucoma dataset are still good but much worse with scores around 0.7. The different possible slot fillers are shown in Table 9 in the Appendix 4. Looking at the different slot fillers, it is not immediately clear why the diabetes case is so much easier for both approaches than the glaucoma case. Both tables have approximately the same number of different entries and in both cases the journal names are in many cases trivial to recognize (containing either Diabetes or Ophthalmol).

However, the distribution of occurrences might partially explain the performance differences here. Although both datasets have similar number of Journal slot fillers with up to three occurrences, only the type 2 diabetes dataset has (even multiple) Journal slot fillers with a high number of occurrences (more than $\approx 8$, e.g.). Therefore, the reason why the Journal slot appears to be so much easier to recognize in the type 2 diabetes dataset might not be due to the textual form of the slot fillers but instead because fewer slot fillers account for a larger majority of the general slot occurrences compared to the glaucoma dataset. The absolute numbers and differences are still quite small, however, but this might allow to get much better scores just by recognizing two or three Journal slot fillers. There may be many more examples which are not discussed here.

All in all it is not clear in all cases what properties of the data cause those partial differences in performance. However, it underlines on the one hand how much data variance can influence information extraction approaches like the two presented ones. On the other hand, this also emphasizes how both approaches can have different strengths and weaknesses and a flat evaluation only considering the final single performance score does not do justice to the complex nature of the task.

### Case study

Similarly to the work by Witte and Cimiano [14], we conduct a case study on a single RCT abstract in which we compare the predicted and ground truth results for one exemplary document out of the type 2 diabetes test dataset. For this case study, we use the same publication as considered by Witte and Cimiano [14] which is the one by Shankar et al. [64]. The results of this case study can be found in Table 8 in the Appendix 3.

Both the extractive and the generative approach succeed in extracting the basic characteristics of the trial which are part of the Publication template, e.g. authors, title and publication year. This is consistent with the results of Table 5, which indicate that Publication is an especially easy template to extract. Similarly, the ClinicalTrial and Medication instances are, except some small errors, extracted almost perfectly. The template instance for the used Intervention is also extracted without errors by both approaches, which is a little more surprising taking into account the slightly lower score of around 0.6. Moreover, both approaches correctly predict that there are no textual slot fillers of the Arm template in the text.

For the Population template instance, we first encounter moderate differences to the gold standard. Although both approaches manage to extract USA as slot fillers for the Country slot, both fail to extract the second slot filler Australia as well as Ethnicity. The latter is at least in line with the fact that the first gold standard precondition - mentioning the ethnicity of the patients - is not recognized by both approaches. For the second Precondition slot filler, both approaches get a part of it but not the full slot filler, with the generative approach recognizing a slightly larger part of the actual slot filler. This is to some degree unexpected, as the mean performance of the extractive approach on the Population templates of the type 2 diabetes dataset is more than twice as high as the score of the generative approach.

For the DiffBetweenGroups template, the extractive approach returns a perfect result in this case, whereas the generative approach misses the $P <0.001$ slot filler but delivers a duplicate of the $P = 0.013$ slot filler. The mean results of Table 5 suggest similar performance, which is not the case here.

For the Endpoint template instances, both approaches manage to extract most slot fillers at least partially but show issues grouping them together correctly. The extractive approach puts all of the extracted slot fillers in just two instances, missing most instances of the gold standard. For the generative approach, however, it is the other way around and too many instances (containing some duplicates) are generated. Nevertheless, some of the generated instances are correct and in some cases there is just a part missing. Generally, the performance is rather unsatisfying here but is consistent with the comparably poor mean performance of around 0.4 on the Endpoint template, indicating this is an especially hard template to extract.

However, the situation is even worse for the Outcome template instances, which was to be expected considering the mean performance on the type 2 diabetes dataset of just 0.2 and 0.11 for the generative and extractive approach, respectively. Again, both approaches at least partially recognize most slot fillers, but fail to group them together correctly. Similarly to the Endpoint template instances, the extractive approach generates too few instances whereas the generative approach generates more instances. Nevertheless, those instances are not entirely correct in most cases. This suggests future work has to improve this grouping beyond simple similarity calculations or fully relying on the language model and constrained decoding.

Taken together, the current results, while promising, are not accurate enough to support the full automatic creation of a systematic review as proposed by Sanchez-Graillet et al. [10]. However, the proposed approach could considerably reduce the workload for teams to extract key information from a set of publications in the sense proposed by Thomas et al. [65]. The results, however, would need to be manually controlled. While the approach is not yet suited to support the full creation of a systematic review at high-quality, it could be used to summarize the existing literature in a cost-effective fashion to allow researchers to get a first overview of existing clinical evidence or as a basis to form hypothesis to be validated further on.

## Conclusion

We have presented an extended extractive and a generative approach for extracting structured information from Randomized Controlled Trial abstracts, which can both support clinicians in finding best therapies on the basis of clinical evidence and in creating systematic reviews of the whole body of available clinical evidence. The extractive approach is realized by a two-step architecture which first extracts slot-fillers from the input document, followed by a clustering step which assigns the extracted slot-fillers to template instances. The best models of this approach yield an average $F_1$ score of 0.547 on type 2 diabetes and 0.636 on glaucoma test sets, respectively. In the generative approach, the structured information given by the template instances is encoded as a linear token sequence which is decoded at inference time by utilizing a context-free grammar for guidance. The best models of the generative approach yield an average $F_1$ score of 0.539 on type 2 diabetes and 0.584 on glaucoma test sets, respectively.

Future work should investigate whether the lead of the extractive approach persists when the base models of both approaches are scaled up, e.g. by using flan-t5-large, flan-t5-xl or even flan-t5-xxl or other large language models. The benefits of the extractive and generative approach could also be combined by adding a pointer network to the generative model. We will also investigate whether integrating a pointer network into the generative model can improve results. It would be also interesting to test the results in an actual evidence generation and comparison case study to assess whether the approach can indeed support the process of summarizing results from the clinical literature for a particular research question.

## Figures and Tables

**Figure:**
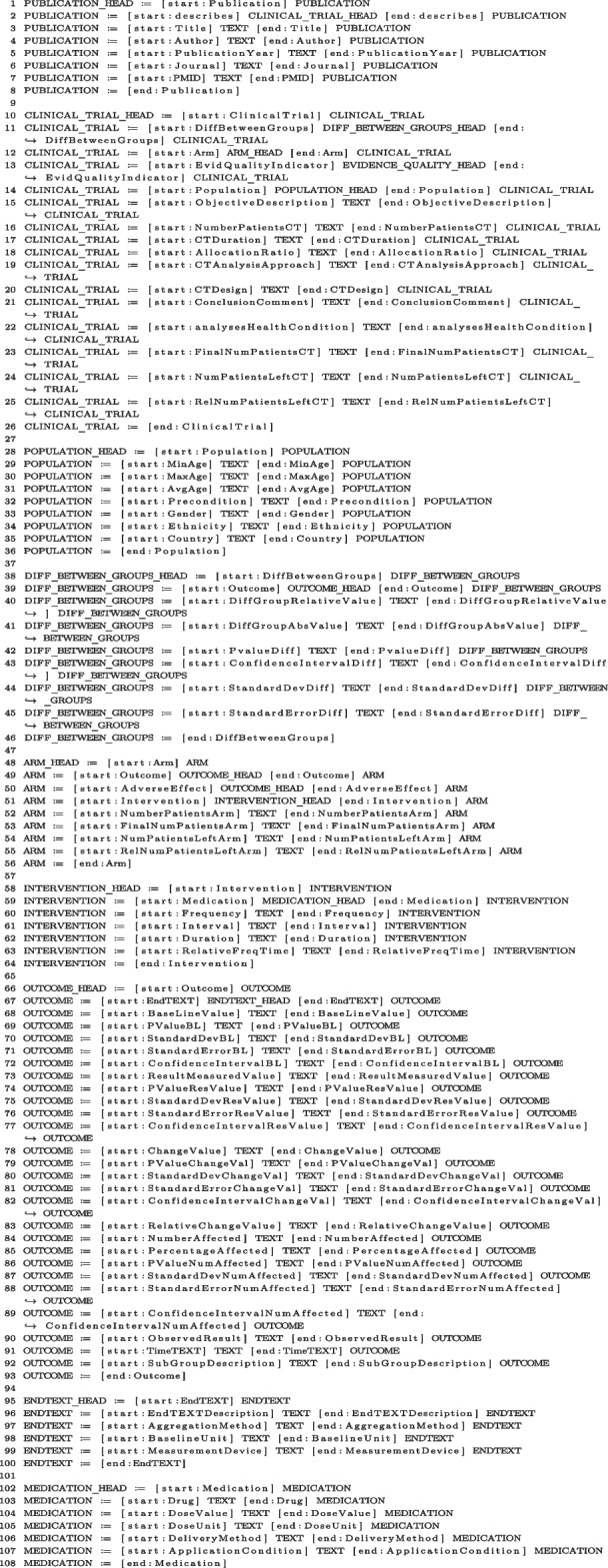
**Listing 1** Grammar of the data model used for decoding in our experiments

**Figure:**
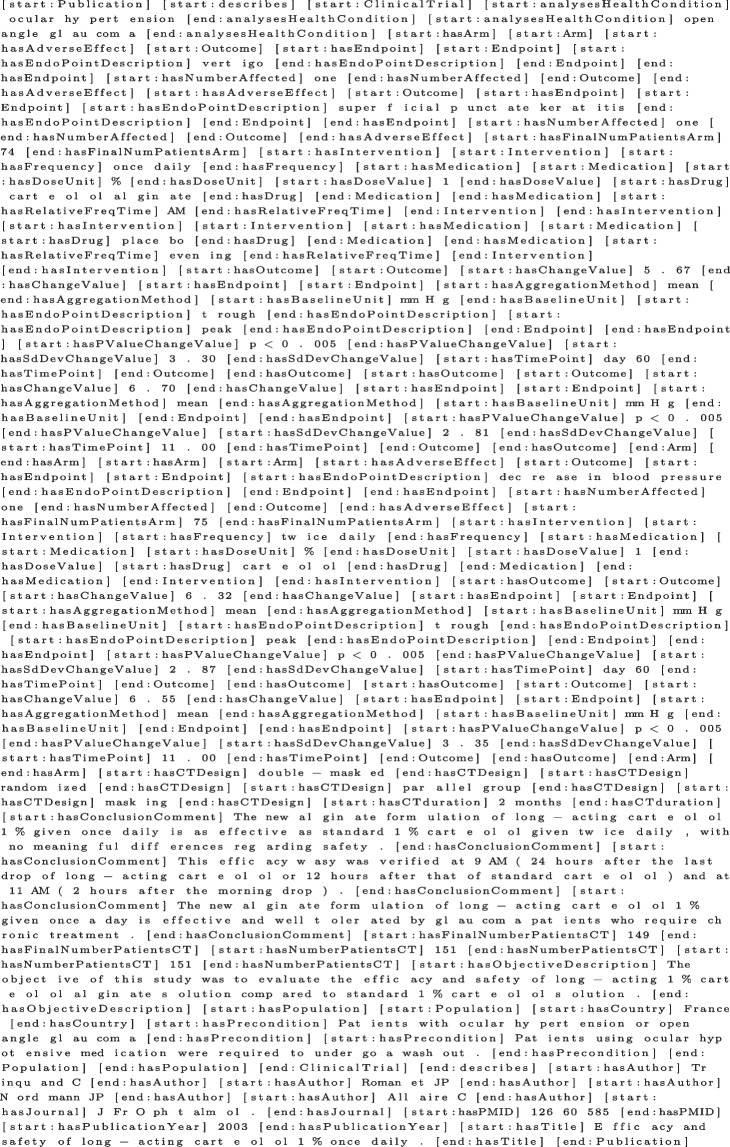
**Listing 2** Linearization of Glaucoma Publication Template Instance

## Data Availability

The code and datasets generated and/or analysed during the current study are available in the Zenodo repository, https://doi.org/10.5281/zenodo.10419786 [66].

## Appendix 2 Slot evaluation

**Table 7:** Mean test $F_1$ scores of the best models of each category per slot (mean and standard deviation of 10 training runs)

	Type 2 diabetes $F_1$		Glaucoma $F_1$	
Slot name	Generative	Extractive	Generative	Extractive
AggregationMethod	0.41 (± 0.07)	0.54 (± 0.03)	0.52 (± 0.08)	0.67 (± 0.13)
AllocationRatio	0.0 (± 0.0)	0.92 (± 0.05)	-	-
analysesHealthCondition	0.84 (± 0.05)	0.73 (± 0.06)	0.87 (± 0.02)	0.86 (± 0.03)
Author	0.97 (± 0.03)	0.92 (± 0.01)	0.8 (± 0.02)	0.94 (± 0.04)
AvgAge	0.0 (± 0.0)	0.37 (± 0.06)	-	-
BaselineUnit	0.42 (± 0.03)	0.44 (± 0.05)	0.54 (± 0.06)	0.56 (± 0.07)
BaselineValue	0.49 (± 0.06)	0.3 (± 0.03)	0.67 (± 0.08)	0.59 (± 0.03)
CTDesign	0.82 (± 0.02)	0.9 (± 0.01)	0.8 (± 0.04)	0.82 (± 0.03)
CTduration	0.89 (± 0.03)	0.89 (± 0.04)	0.78 (± 0.06)	0.87 (± 0.04)
ChangeValue	0.41 (± 0.06)	0.19 (± 0.05)	0.59 (± 0.07)	0.52 (± 0.05)
ConclusionComment	0.73 (± 0.04)	0.88 (± 0.04)	0.84 (± 0.02)	0.91 (± 0.02)
ConfIntervalChangeValue	0.0 (± 0.0)	0.0 (± 0.0)	-	-
ConfIntervalDiff	0.46 (± 0.12)	0.43 (± 0.05)	0.29 (± 0.11)	0.28 (± 0.11)
Country	0.68 (± 0.07)	0.64 (± 0.08)	0.86 (± 0.05)	0.86 (± 0.03)
DeliveryMethod	0.0 (± 0.0)	0.0 (± 0.0)	0.34 (± 0.2)	0.42 (± 0.06)
DiffGroupAbsValue	0.45 (± 0.09)	0.43 (± 0.06)	0.31 (± 0.16)	0.43 (± 0.11)
DoseDescription	0.0 (± 0.0)	0.0 (± 0.0)	-	-
DoseUnit	0.77 (± 0.04)	0.24 (± 0.04)	0.8 (± 0.08)	0.6 (± 0.06)
DoseValue	0.79 (± 0.04)	0.77 (± 0.07)	0.75 (± 0.07)	0.65 (± 0.06)
Drug	0.82 (± 0.05)	0.7 (± 0.02)	0.58 (± 0.04)	0.45 (± 0.06)
Duration	-	-	0.0 (± 0.0)	0.2 (± 0.32)
EndoPointDescription	0.34 (± 0.02)	0.3 (± 0.02)	0.26 (± 0.05)	0.25 (± 0.03)
FinalNumPatientsArm	0.0 (± 0.0)	-	0.0 (± 0.0)	0.02 (± 0.06)
FinalNumberPatientsCT	-	-	0.0 (± 0.0)	0.64 (± 0.13)
Frequency	0.61 (± 0.06)	0.62 (± 0.02)	0.77 (± 0.06)	0.71 (± 0.04)
Journal	0.96 (± 0.05)	0.92 (± 0.05)	0.67 (± 0.08)	0.74 (± 0.07)
MeasurementDevice	-	-	0.0 (± 0.0)	0.2 (± 0.32)
MinAge	0.0 (± 0.0)	0.67 (± 0.17)	-	-
NumberAffected	0.16 (± 0.19)	0.08 (± 0.13)	0.4 (± 0.22)	0.0 (± 0.0)
NumberPatientsArm	0.83 (± 0.09)	0.87 (± 0.02)	0.68 (± 0.12)	0.7 (± 0.06)
NumberPatientsCT	0.65 (± 0.08)	0.93 (± 0.04)	0.65 (± 0.09)	0.86 (± 0.02)
ObjectiveDescription	0.43 (± 0.06)	0.49 (± 0.05)	0.49 (± 0.07)	0.51 (± 0.09)
ObservedResult	0.03 (± 0.03)	0.01 (± 0.01)	0.01 (± 0.03)	0.0 (± 0.0)
PMID	0.97 (± 0.03)	1.0 (± 0.0)	0.98 (± 0.02)	0.99 (± 0.01)
PValueChangeValue	0.1 (± 0.11)	0.0 (± 0.0)	0.0 (± 0.0)	0.33 (± 0.05)
PercentageAffected	0.59 (± 0.08)	0.21 (± 0.03)	0.31 (± 0.18)	0.15 (± 0.03)
Precondition	0.22 (± 0.08)	0.4 (± 0.07)	0.27 (± 0.05)	0.18 (± 0.04)
PublicationYear	0.97 (± 0.03)	1.0 (± 0.0)	0.98 (± 0.02)	1.0 (± 0.0)
PvalueDiff	0.31 (± 0.05)	0.48 (± 0.02)	0.24 (± 0.06)	0.39 (± 0.06)
RelativeChangeValue	0.04 (± 0.09)	0.0 (± 0.0)	0.13 (± 0.2)	0.57 (± 0.12)
RelativeFreqTime	-	-	0.0 (± 0.0)	0.36 (± 0.1)
ResultMeasuredValue	0.27 (± 0.07)	0.21 (± 0.04)	0.57 (± 0.07)	0.35 (± 0.02)
SdDevBL	0.18 (± 0.18)	0.14 (± 0.19)	0.53 (± 0.09)	0.62 (± 0.05)
SdDevChangeValue	0.02 (± 0.06)	0.0 (± 0.0)	0.38 (± 0.14)	0.45 (± 0.07)
SdDevResValue	0.2 (± 0.13)	0.19 (± 0.02)	0.62 (± 0.13)	0.34 (± 0.02)
SdErrorChangeValue	-	-	0.0 (± 0.0)	0.57 (± 0.0)
SubGroupDescription	0.0 (± 0.0)	0.0 (± 0.0)	-	-
TimePoint	0.35 (± 0.11)	0.22 (± 0.03)	0.39 (± 0.07)	0.41 (± 0.03)
Title	0.86 (± 0.05)	0.93 (± 0.02)	0.88 (± 0.06)	0.85 (± 0.03)
Total Micro $F_1$ Score	0.54 (± 0.03)	0.55 (± 0.01)	0.58 (± 0.02)	0.64 (± 0.01)

## Appendix 3 Case study

**Table 8:** Case study for disease Type 2 Diabetes. Multiple entries for same slot in same template instance separated by |

Slot name	Gold standard	Extractive prediction	Generative prediction
**Template Publication**			
describes			
Author	Shankar RR | Bao Y | Han P | Hu J | Ma J | Peng Y | Wu F | Xu L | Engel SS | Jia W	Shankar RR | Bao Y | Han P | Hu J | Ma J | Peng Y | Wu F | Xu L | Engel SS | Jia W	Shankar RR | Bao Y | Han P | Hu J | Ma J | Peng Y | Wu F | Xu L | Engel SS | Jia W
Journal	J Diabetes Investig.	J Diabetes Investig.	J Diabetes Investig.
PMID	27740719	27740719	27740719
PublicationYear	2017	2017	2017
Title	Sitagliptin added to stable insulin therapy with or without metformin in Chinese patients with type 2 diabetes.	Sitagliptin added to stable insulin therapy with or without metformin in Chinese patients with type 2 diabetes.	Sitagliptin added to stable insulin therapy with or without metformin in Chinese patients with type 2 diabetes.
**Template Intervention**			
Frequency	once daily	once daily	once daily
Medication			
**Template Population**			
Country	USA | Australia	USA	USA
Ethnicity	Chinese		
Precondition	Chinese patients with type 2 diabetes mellitus receiving stable insulin therapy alone or in combination with metformin | patients with inadequate glycemic control on insulin ( glycated hemoglobin [ HbA1c ] < 7. 5 % and < 11 % )	patients with inadequate glycemic control on insulin	patients with inadequate glycemic control on insulin ( glycated hemoglobin [ HbA1c ] <
**Template Arm**			
AdverseEffect			
Intervention			
Outcome			
**Template Endpoint**			
BaselineUnit	%	%	
EndoPointDescription	HbA1c	HbA1c of <7. 0 %	adverse events
BaselineUnit		mg / dL | mg / dL	
EndoPointDescription	HbA1c of < 7. 0 %	fasting plasma glucose | 2 - h post - meal glucose | adverse events of hypoglycemia	hypoglycemia
BaselineUnit	mg / dL		%
EndoPointDescription	2 - h post - meal glucose		HbA1c
BaselineUnit	mg / dL		
EndoPointDescription	fasting plasma glucose		HbA1c of <
BaselineUnit			mg / dL
EndoPointDescription	hypoglycemia ( symptomatic or asymptomatic )		2 - h post - meal glucose
BaselineUnit			
EndoPointDescription	bodyweight		fasting plasma glucose
BaselineUnit			
EndoPointDescription			adverse events
BaselineUnit			
EndoPointDescription			hypoglycemia
BaselineUnit			%
EndoPointDescription			HbA1c
BaselineUnit			
EndoPointDescription			HbA1c of <
BaselineUnit			mg / dL
EndoPointDescription			2 - h post - meal glucose
BaselineUnit			
EndoPointDescription			fasting plasma glucose
**Template ClinicalTrial**			
analysesHealthCondition	type 2 diabetes mellitus	type 2 diabetes	type 2 diabetes mellitus
AllocationRatio	1 : 1	1 : 1	
Arm			
CTDesign	randomized	randomized	randomized
CTduration	24 weeks	24 weeks	24 weeks
ConclusionComment	After 24 weeks, sitagliptin added to stable insulin therapy ( < metformin ) was generally well tolerated and improved glycemic control in Chinese patients with type 2 diabetes mellitus.	After 24 weeks, sitagliptin added to stable insulin therapy ( <metformin ) was generally well tolerated and improved glycemic control in Chinese patients with type 2 diabetes mellitus.	After 24 weeks, sitagliptin added to stable insulin therapy ( < | ) was generally well tolerated and improved glycemic control in Chinese patients with type 2 diabetes mellitus.
DiffBetweenGroups			
NumberPatientsCT	467	467	467
ObjectiveDescription	We evaluated the tolerability and efficacy of the addition of sitagliptin in Chinese patients with type 2 diabetes mellitus receiving stable insulin therapy alone or in combination with metformin.	We evaluated the tolerability and efficacy of the addition of sitagliptin in Chinese patients with type 2 diabetes mellitus receiving stable insulin therapy alone or in combination with metformin.	We evaluated the tolerability and efficacy of the addition of sitagliptin in Chinese patients with type 2 diabetes mellitus receiving stable insulin therapy alone or in combination with metformin.
Population			
**Template Medication**			
DoseUnit	mg		mg
DoseValue	100	100	100
Drug	sitagliptin	sitagliptin	sitagliptin
DoseUnit		mg	
DoseValue			
Drug	placebo	placebo	placebo
**Template Outcome**			
ChangeValue	0. 7	0. 7	
Endpoint			
NumberAffected			64
ObservedResult			
PercentageAffected			27. 4
TimePoint		week 24	
ChangeValue	0. 3	26. 5 | 0. 3 | 14. 4	
Endpoint			
NumberAffected			51
ObservedResult			
PercentageAffected		16 | 21. 9 | 64 | 8 | 27. 4 | 51	21. 9
TimePoint		week 24	
ChangeValue			0. 7
Endpoint			
NumberAffected			
ObservedResult			
PercentageAffected	16		
TimePoint	week 24		week 24
ChangeValue			
Endpoint			
NumberAffected			
ObservedResult			
PercentageAffected	8		16
TimePoint	week 24		week 24
ChangeValue	26. 5		
Endpoint			
NumberAffected			
ObservedResult			
PercentageAffected			
TimePoint			
ChangeValue	14. 4		
Endpoint			
NumberAffected			51
ObservedResult			
PercentageAffected			21. 9
TimePoint			
ChangeValue	10. 7		
Endpoint			
NumberAffected			51
ObservedResult			
PercentageAffected			21. 9
TimePoint			
ChangeValue			0. 3
Endpoint			
NumberAffected	64		
ObservedResult			
PercentageAffected	27. 4		
TimePoint			week 24
ChangeValue			
Endpoint			
NumberAffected	51		
ObservedResult			
PercentageAffected	21. 9		8
TimePoint			week 24
ChangeValue			
Endpoint			
NumberAffected			
ObservedResult	Neither group had a significant change from baseline in bodyweight.		
PercentageAffected			
TimePoint			
**Template DiffBetweenGroups**			
Outcome1			
Outcome2			
PvalueDiff	P < 0. 001	P <0. 001	P = 0. 013
Outcome1			
Outcome2			
PvalueDiff	P = 0. 013	P = 0. 013	P = 0. 013
Outcome1			
Outcome2			
PvalueDiff	P < 0. 001	P <0. 001	

## Appendix 4 Journal slot fillers

**Table 9:** Slot fillers of slot Journal with number of occurrences in the type 2 diabetes dataset and glaucoma training and test datasets

Type 2 diabetes	#	Glaucoma	#
‘Ann Intern Med .’	2	‘Acta Ophthalmol .’	1
‘Arch Intern Med .’	1	‘Acta Ophthalmol Scand .’	3
‘Arch Med Res .’	1	‘Acta Ophthalmol Scand’	2
‘BMC Endocr Disord .’	1	‘Acta Ophthalmol’	1
‘Cardiovasc Diabetol .’	1	‘Adv Ther .’	2
‘Clin Drug Investig .’	1	‘Am J Ophthalmol .’	4
‘Clin Ther .’	4	‘Am J Ophthalmol’	2
‘Curr Med Res Opin .’	6	‘Arch Ophthalmol .’	3
‘Diabet Med .’	6	‘BMC Ophthalmol .’	2
‘Diabetes Care .’	2	‘Br J Ophthalmol .’	5
‘Diabetes Care’	12	‘Br J Ophthalmol’	1
‘Diabetes Metab Res Rev .’	2	‘Clin Ophthalmol’	1
‘Diabetes Obes Metab .’	19	‘Clin Ther .’	4
‘Diabetes Res Clin Pract .’	1	‘Clin Ther’	1
‘Diabetes Technol Ther .’	2	‘Coll Antropol’	1
‘Diabetes’	1	‘Curr Med Res Opin .’	5
‘Diabetologia’	3	‘Curr Med Res Opin’	1
‘Endocr J .’	1	‘Curr Ther Res Clin Exp .’	1
‘Exp Clin Endocrinol Diabetes’	1	‘Eur J Ophthalmol .’	2
‘Horm Metab Res .’	3	‘Eur J Ophthalmol’	1
‘Horm Res .’	1	‘Eye ( Lond ) .’	6
‘J Diabetes Complications’	2	‘Eye ( Lond )’	1
‘J Diabetes Investig .’	1	‘Graefes Arch Clin Exp Ophthalmol .’	4
‘J Diabetes’	3	‘Int Ophthalmol .’	1
‘Lancet’	2	‘Invest Ophthalmol Vis Sci .’	1
‘Med J Malaysia’	1	‘J Fr Ophtalmol .’	1
‘Obesity ( Silver Spring )’	1	‘J Glaucoma’	9
‘Open Access Maced J Med Sci .’	1	‘J Ocul Pharmacol .’	1
‘PLoS One’	1	‘J Ocul Pharmacol Ther .’	5
‘Pediatr Diabetes’	1	‘J Ocul Pharmacol Ther’	2
‘Pharmacotherapy’	1	‘Jpn J Ophthalmol’	2
‘Srp Arh Celok Lek .’	1	‘Lancet’	1
		‘Nippon Ganka Gakkai Zasshi .’	1
		‘Ophthalmology’	8
		‘Saudi Med J’	1
		‘Surv Ophthalmol .’	1

## Appendix 5 Template cardinalities

**Table 10:** Cardinality Evaluation Type 2 Diabetes Generative

Template name	Mean GT count	Mean predicted count	Abs diff
Arm	2.00	1.99 (± 0.02)	0.01 (± 0.02)
DiffBetweenGroups	2.75	3.78 (± 0.95)	1.01 (± 0.96)
Endpoint	5.85	11.16 (± 1.11)	5.32 (± 1.11)
EvidenceQuality	1.00	0.0 (± 0.0)	1.0 (± 0.0)
Intervention	2.10	2.0 (± 0.04)	0.1 (± 0.04)
Medication	2.20	1.99 (± 0.07)	0.21 (± 0.07)
Outcome	10.35	11.16 (± 1.11)	1.08 (± 0.82)

**Table 11:** Cardinality Evaluation Type 2 Diabetes Extractive

Template name	Mean GT count	Mean predicted count	Abs diff
Arm	2.00	0.91 (± 0.03)	1.09 (± 0.03)
DiffBetweenGroups	2.75	2.06 (± 0.08)	0.69 (± 0.08)
Endpoint	5.85	2.02 (± 0.03)	3.83 (± 0.03)
EvidenceQuality	1.00	0.0 (± 0.0)	1.0 (± 0.0)
Intervention	2.10	0.88 (± 0.03)	1.22 (± 0.03)
Medication	2.20	2.02 (± 0.1)	0.18 (± 0.1)
Outcome	10.35	1.99 (± 0.07)	8.36 (± 0.07)

**Table 12:** Cardinality Evaluation Glaucoma Generative

Template name	Mean GT count	Mean predicted count	Abs diff
Arm	2.00	1.98 (± 0.04)	0.02 (± 0.04)
DiffBetweenGroups	1.62	1.99 (± 0.38)	0.39 (± 0.36)
Endpoint	2.48	5.39 (± 1.09)	2.91 (± 1.09)
EvidenceQuality	1.00	0.0 (± 0.0)	1.0 (± 0.0)
Intervention	2.19	2.0 (± 0.05)	0.19 (± 0.05)
Medication	2.33	2.31 (± 0.14)	0.11 (± 0.08)
Outcome	5.05	5.39 (± 1.09)	0.94 (± 0.57)

**Table 13:** Cardinality Evaluation Glaucoma Extractive

Template name	Mean GT count	Mean predicted count	Abs diff
Arm	2.00	0.67 (± 0.05)	1.33 (± 0.05)
DiffBetweenGroups	1.70	1.85 (± 0.16)	0.17 (± 0.13)
Endpoint	2.48	2.13 (± 0.22)	0.35 (± 0.22)
EvidenceQuality	1.00	0.0 (± 0.0)	1.0 (± 0.0)
Intervention	2.19	1.39 (± 0.1)	0.8 (± 0.1)
Medication	2.33	1.86 (± 0.2)	0.47 (± 0.2)
Outcome	5.05	2.07 (± 0.04)	2.98 (± 0.04)

## Abbreviations

BERT: Bidirectional encoder representations from transformers
C-TrO: Clinical trial ontology; CFG: context-free grammar
CNN: Convolutional neural network
CRF: Conditional random field
EBM: Evidence-based medicine
Flan: Finetuning language models
GenIE: Generative information extraction
GT: Ground truth
HAC: Hierarchical agglomerative clustering
IE: Information extraction
iff: if and only if
ITC: Intra-template compatibility
LED: Longformer-encoder-decoder
LSTM: Long short-term memory network
MAD: Mean absolute deviation
MAP: Maximum a posteriori probability
PICO: Patient, intervention, comparison, outcomes
RCT: Randomized controlled trial
REBEL: Relation extraction by end-to-end language generation
ReLU: Rectified linear unit
SF: Slot-filler
T5: Text-to-text transfer transformer
TI: Template instance
